# Rapid intracerebroventricular delivery of Cu-DOTA-etanercept after peripheral administration demonstrated by PET imaging

**DOI:** 10.1186/1756-0500-2-28

**Published:** 2009-02-27

**Authors:** Edward L Tobinick, Kai Chen, Xiaoyuan Chen

**Affiliations:** 1 Institute for Neurological Research, a private medical group, inc., 100 UCLA Medical Plaza, Suites 205-210, Los Angeles, California 90095 USA; 2 Stanford University School of Medicine, 1201 Welch Rd, P087, Stanford, California 94305-5484 USA

## Abstract

**Background:**

The cytokines interleukin-1 and tumor necrosis factor (TNF), and the cytokine blocker interleukin-1 receptor antagonist, all have been demonstrated to enter the cerebrospinal fluid (CSF) following peripheral administration. Recent reports of rapid clinical improvement in patients with Alzheimer's disease and related forms of dementia following perispinal administration of etanercept, a TNF antagonist, suggest that etanercept also has the ability to reach the brain CSF. To investigate, etanercept was labeled with a positron emitter to enable visualization of its intracranial distribution following peripheral administration by PET in an animal model.

**Findings:**

Radiolabeling of etanercept with the PET emitter ^64^Cu was performed by DOTA (1,4,7,10-tetraazadodecane-N,N',N",N"'-tetraacetic acid) conjugation of etanercept, followed by column purification and ^64^Cu labeling. MicroPET imaging revealed accumulation of ^64^Cu-DOTA-etanercept within the lateral and third cerebral ventricles within minutes of peripheral perispinal administration in a normal rat anesthesized with isoflurane anesthesia, with concentration within the choroid plexus and into the CSF.

**Conclusion:**

Synthesis of ^64^Cu-DOTA-etanercept enabled visualization of its intracranial distribution by microPET imaging. MicroPET imaging documented rapid accumulation of ^64^Cu-DOTA-etanercept within the choroid plexus and the cerebrospinal fluid within the cerebral ventricles of a living rat after peripheral administration. Further study of the effects of etanercept and TNF at the level of the choroid plexus may yield valuable insights into the pathogenesis of Alzheimer's disease.

## Background

Excess tumor necrosis factor-alpha (TNF) has been identified as a therapeutic target in Alzheimer's disease (AD)[1]. Excess TNF in the cerebrospinal fluid (CSF), at concentrations 25 times higher than in controls, has been demonstrated in AD, and may predict disease progression[2]. Etanercept is a recombinant dimeric fusion protein consisting of the extracellular ligand-binding portions of two human p75 TNF receptors linked to the Fc fragment of human IgG1, which functions *in vivo *as a potent anti-TNF therapeutic. Recent reports of rapid clinical improvement in patients with AD and related disorders following the perispinal administration of etanercept (MW = 150,000) suggested that etanercept had the ability to penetrate into the CSF in the brain in a therapeutically effective concentration[3,4], an ability which recently had been demonstrated for another cytokine antagonist, interleukin-1 receptor antagonist (IL1-RA) (MW = 17,000)[5]. To investigate this possibility, etanercept was conjugated with ^64^Cu, using a method developed by one of the authors [6]. ^64^Cu is a positron emitter. When attached to etanercept, utilizing the chelating agent (1,4,7,10-tetraazadodecane-N,N',N",N"'-tetraacetic acid (DOTA), a molecule is produced, ^64^Cu-DOTA-etanercept, whose anatomic distribution may be imaged using positron emission tomography (PET)[6]. To examine the intracranial distribution of radiolabeled etanercept, microPET imaging of the brain of a living rat following peripheral administration of ^64^Cu-DOTA-etanercept was performed.

## Methods

Animal studies were conducted in accordance with the applicable protocols by the Stanford Animal Care Committee. Etanercept (Immmunex, Amgen) was commercially purchased in powder form. Preparation of ^64^Cu-(1,4,7,10-tetraazadodecane-N,N',N",N"'-tetraacetic acid (DOTA)-etanercept was as previously described[6]. 150 microliters of ^64^Cu-DOTA-etanercept solution (ca. 1 mCi) was injected overlying the cervical spine of a 250 g Sprague-Dawley rat at the C 6–7 level using a 30 gauge needle at a depth of 6 mm while the rat was anesthetized with 2.5% isoflurane inhalation anesthesia. The rat was then placed in the head down position by tail suspension for three minutes, immediately followed by placement horizontally in the bed of a microPET imaging scanner (microPET R4 rodent model scanner, Siemens Medical Solutions USA, Inc.) designed for 5-min static scans; the scan was initated two minutes after placement in the scanner bed and was performed from five to ten minutes after etanercept administration. The rationale for this method of peripheral administration is to deliver etanercept into the cerebrospinal venous system, as previously discussed[3,4,7-9]. The images were reconstructed by a 2-dimensional ordered-subsets expectation maximum (OSEM) algorithm, and no correction was necessary for attenuation or scatter correction.

## Results

**Figure 1:**
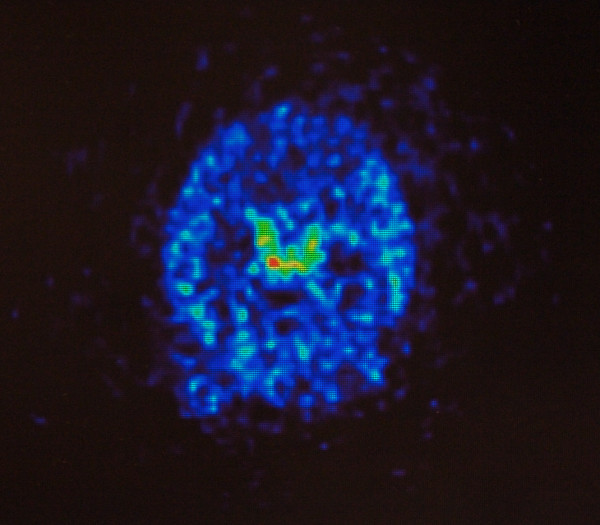
**PET image, transverse section, of a living rat brain following perispinal extrathecal administration of ^64^Cu-DOTA-etanercept, imaged 5 to 10 minutes following etanercept administration**. The pattern is consistent with penetration of ^64^Cu-DOTA-etanercept into the cerebrospinal fluid in the lateral and third ventricles. Note the horizontal linear enhancement within the lateral ventricles which is suggestive of accumulation of tracer within the choroid plexus.

MicroPET imaging revealed accumulation of ^64^Cu-DOTA-etanercept within the lateral and third cerebral ventricles within minutes of peripheral perispinal administration, with concentration within the choroid plexus and into the CSF suggested by the microPET images (Figure 1). These non-invasive PET results are quite analagous to the results of previous autoradiographic studies utilizing [^125^I] labeled TNF, IL-1, and IL1-RA which demonstrated CSF penetration within the cerebral ventricles in mice following peripheral administration of each of these large molecules[10].

## Discussion

The apparent ability of etanercept to traverse the BCSFB and enter the CSF in the brain demonstrated in this experiment suggests that etanercept joins interleukin 1-RA (IL1-RA), interleukin-1 (IL-1) (MW = 17,000), and TNF (MW = 17,000 as a monomer; 51,000 as a soluble trimer) as large molecules which penetrate into the cerebrospinal fluid in the brain after peripheral delivery[5,10,11]. These findings may have significant implications for the treatment of certain brain disorders, including the use of IL1-RA for the treatment of stroke and the use of etanercept for the treatment of Alzheimer's disease[1,3-5,7].

Rapid delivery of etanercept into the CSF within the cerebral ventricles following peripheral delivery was subsequently confirmed in a separate, later experiment in multiple rats performed at the University of British Columbia in Vancouver begun several months following the completion of this study performed at Stanford(manuscript in preparation)[1].

These results highlight the important functional differences between the blood-cerebrospinal fluid barrier (BCSFB) and the blood-brain barrier (BBB)[12,13]. The BBB, formed by tight junctions between the endothelial cells of the brain capillaries, prevents the passage of essentially all large molecules, i.e. those with a molecular weight (MW) greater than approximately 500 daltons[14]. The BCSFB, formed primarily by the epithelial cells of the choroid plexus, appears to be more permeable with respect to large molecules than the BBB[13]. Thus IL1-RA, IL-1, TNF, and etanercept plausibly all enter the CSF by traversing the BCSFB via the choroid plexus[1,10,11]. Failure to image the cerebral ventricles by either autoradiography or by using PET imaging, as in the present experiment(Figure 1), may have led, in one study[15], to a failure to detect delivery of large molecules into the cerebrospinal fluid and the choroid plexus.

Delivery of etanercept to the choroid plexus (CP) may have significant physiologic implications. The CP is the source of synthesis of numerous molecules, including cytokines and growth factors, including TNF, TGF-alpha, TGF-beta, FGF2 and IGF-II[12,13,16]. Epithelial cells of the choroid plexus are known to express TNF[17]. The CP has been postulated to be centrally involved in the pathogenesis of AD[12,13,16]. It is possible that etanercept reaching the CP may have intrinsic effects on CP cellular function and cytokine and growth factor synthesis. Etanercept reaching the CP could have paracrine or autocrine effects on the CP, or potentially could affect the parenchyma in the periventricular or additional brain regions by endocrine-like bulk flow of CSF[12,13]. Additionally, etanercept reaching the CP and the CSF could have glial effects[1]. Alteration of glial modulation of neuronal function mediated by TNF, beginning within the CP, could potentially produce widespread neuronal and cortical effects[1,3,4].

The effect of intracerebroventricular delivery of anti-TNF biologics has recently been examined in two experimental models investigating AD mechanisms[18,19]. In the first study, intracerebroventricular delivery of infliximab, an anti-TNF monoclonal antibody, prevented the inhibition of LTP at hippocampal CA1 synapses caused by intracerebroventicular injection of beta-amyloid [18]. In the second study, intracerebroventricular delivery of an anti-TNF monoclonal antibody prevented the nitration of proteins in the hippocampus and the impairment of recognition memory in mice induced by beta-amyloid fragments[19]. The results of these additional experimental models, along with the known involvement of the CP in cytokine synthesis, suggest that rapid delivery of ^64^Cu-DOTA-etanercept into the choroid plexus may provide a potential explanation for the rapid clinical improvement noted following perispinal administration of etanercept in AD[1,3,4]. TNF is known to be capable of producing synaptic effects within minutes[20].

Further investigation regarding the mechanisms by which etanercept enters the cerebral ventricles, presumably by crossing the BCSFB are warranted. At this time the exact mechanism by which this occurs at the CP is unknown. At the CP, in addition to specific saturable transport systems for selected large molecules, there also exists a less known paracellular diffusion pathway, which hydrophilic solutes may utilize to penetrate the CSF, diffusing between choroidal epithelial cells rather than through them[13]. Intravascularly administered inulin polysaccaride (MW = 5,500), for example, has been demonstrated to reach the CSF by this paracellular diffusion pathway across the BCSFB at the CP[13,21,22]. Alternatively, it is possible that etanercept is actively transported across the CP epithelium. One may hypothesize that this could occur via a "piggyback" mechanism, associated with the binding of etanercept to TNF, while TNF is itself actively transported across the CP epithelium[10,11]. This "piggyback" mechanism might be facilitated by the natural occurrence of circulating trimers of TNF which would present more than one binding site per ligand. This speculation will require further study for definitive answers to emerge.

Additional literature supports the argument that the choroid plexus and nearby ependymal regions may be points of entry of macromolecules into the CSF, particularly after head-down tilt. This literature includes the demonstration that head-down tilt, even for as short as five minutes, disrupts the blood-CSF barrier of rabbits, allowing trypan blue to penetrate the CNS[23]; that choroid plexus proteins are expressed/localized in the ventricle-facing apical membrane and choroidal CSF production is increased, shortly after head-down tilt accomplished by hindlimb-suspension in rats[24]; and that macromolecules may gain access to the brain and CSF by extracellular (non-BBB) routes[25]. It has previously been hypothesized that macromolecules, such as antibodies directed against amyloid beta protein and erythropoietin, may exert CNS effects by passage into the brain via extracellular pathways[25]. Etanercept is highly potent, and may have significant physiologic effects at low concentration[26]. Further study will be necessary to clarify if etanercept distribution via extracellular pathways, in addition to effects at the choroid plexus and in the CSF, contributes to the physiologic effects observed after perispinal etanercept administration in conditions such as Alzheimer's disease.

With respect to extrapolation of the results of the present experiment to AD, an additional consideration is necessary. The experimental model used in the present study included only normal animals, but changes in CP physiology and barrier function may accompany both aging and AD, and these changes could further influence the passage of etanercept across the BCSFB[12,13,16,27]. In the clinical studies of perispinal etanercept in AD, Trendelenburg head-down positioning is utilized following cervical perispinal injection, hypothesized to increase access of etanercept to the choroid plexus via the cerebrospinal venous system[1,4,9]. It is hypothesized that head-down positioning, by increasing venous pressure in the CP, may have the potential to influence transport across the BCSFB or across the ependyma or the circumventricular organs into the periventricular brain parenchyma[1,4,23,28]. Effects of tail suspension on ependymal ultrastructure have recently been reported[29]. Further study is needed to confirm this hypothesis.

The limited time of brain imaging, 10 minutes after etanercept administration in this study, does not allow a definitive statement regarding later parenchymal delivery. Future studies will reveal this. It is known that substances which reach the intraventricular cerebrospinal fluid often reach the periventricular brain parenchyma, including the hippocampus, so eventual parenchymal delivery cannot be ruled out by these results[12,13,25]. Macromolecules reaching the CSF characteristically penetrate into periventricular brain parenchyma because the gap junctions between the ependymal cells lining the ventricles are "leaky", thus the popularity of intracerebroventricular delivery to bypass both the BBB and the BCSFB[12,13,30]. Alternatively, it is likely that some of the physiologic effects of perispinal etanercept are mediated primarily at the level of the choroid plexus. Further study will be necessary to define the sites of action of etanercept in the AD brain, the effects of etanercept on the choroid plexus in AD, and to characterize the brain parenchymal distribution of etanercept after CSF delivery.

## Abbreviations

AD: Alzheimer's disease; BBB: blood-brain barrier; BCSFB: blood-cerebrospinal fluid barrier; CP: choroid plexus; CSF: cerebrospinal fluid; DOTA: (1,4,7,10-tetraazadodecane-N,N',N",N"'-tetraacetic acid); FGF2: basic fibroblast growth factor2; IGF-II: insulin-like growth factor II; IL-1: Interleukin-1; IL-1RA: Interleukin-1 receptor antagonist; KD: kilodalton; MW: molecular weight; PET: positron emission tomography; TGF-alpha: transforming growth factor-alpha; TGF-beta: transforming growth factor-beta; TNF: tumor necrosis factor-alpha.

## Competing interests

Author Edward Tobinick has multiple issued and pending patents, assigned to TACT IP LLC, which describe the parenteral and perispinal use of etanercept for the treatment of Alzheimer's disease and other neurological disorders, including, but not limited to, U.S. patents 6015557, 6177077, 6419934, 6419944, 6537549, 6982089, 7214658 and Australian patent 758523. He owns stock in Amgen, the manufacturer of etanercept. In addition, he has pending patents which describe the use of the cerebrospinal venous system and/or perispinal administration to deliver other therapeutic or diagnostic agents to the brain, eye, spinal cord, and other anatomic structures. The additional authors have no competing interests.

## Authors' contributions

All authors read and approved the final manuscript. ET drafted the manuscript, conceived and participated in the design of the study, and assisted in the performance of the animal study. KC participated in the performance of the animal study, including the image acquistion and analysis, and contributed to the drafting of the final version of the manuscript. XC developed the method of radiolabeling etanercept used in the study, participated in the design of the study, participated in the performance of the animal study, performed the image acquistion and analysis, and participated in the drafting of the final version of the manuscript.

## Authors information

Edward Tobinick MD is an Assistant Clinical Professor of Medicine at the David Geffen School of Medicine at UCLA. Xiaoyuan Chen, Ph.D. is an Associate Professor(Research) in the Department of Radiology at Stanford University School of Medicine. Kai Chen, Ph.D. is a post-doctoral fellow in the Molecular Imaging Program at Stanford University School of Medicine.
